# Time-frequency analysis of short-lasting modulation of EEG induced by TMS during wake, sleep deprivation and sleep

**DOI:** 10.3389/fnhum.2013.00767

**Published:** 2013-11-18

**Authors:** Paolo Manganotti, Emanuela Formaggio, Alessandra Del Felice, Silvia F. Storti, Alessandro Zamboni, Alessandra Bertoldo, Antonio Fiaschi, Gianna M. Toffolo

**Affiliations:** ^1^Clinical Neurophysiology and Functional Neuroimaging Unit, Section of Neurology, Department of Neurological and Movement Sciences, University of VeronaVerona, Italy; ^2^Department of Neurophysiology, Foundation IRCCS San Camillo HospitalVenice, Italy; ^3^Department of Information Engineering, University of PadovaPadova, Italy

**Keywords:** EEG, TMS, cortical excitability, wavelet analysis, vigilance state

## Abstract

The occurrence of dynamic changes in spontaneous electroencephalogram (EEG) rhythms in the awake state or sleep is highly variable. These rhythms can be externally modulated during transcranial magnetic stimulation (TMS) with a perturbation method to trigger oscillatory brain activity. EEG-TMS co-registration was performed during standard wake, during wake after sleep deprivation and in sleep in six healthy subjects. Dynamic changes in the regional neural oscillatory activity of the cortical areas were characterized using time-frequency analysis based on the wavelet method, and the modulation of induced oscillations were related to different vigilance states. A reciprocal synchronizing/desynchronizing effect on slow and fast oscillatory activity was observed in response to focal TMS after sleep deprivation and sleep. We observed a sleep-related slight desynchronization of alpha mainly over the frontal areas, and a widespread increase in theta synchronization. These findings could be interpreted as proof of the interference external brain stimulation can exert on the cortex, and how this could be modulated by the vigilance state. Potential clinical applications may include evaluation of hyperexcitable states such as epilepsy or disturbed states of consciousness such as minimal consciousness.

## INTRODUCTION

Oscillatory human brain activity occurs at different frequencies (Niedermeyer, 1999) and can be rapidly modulated over the occipital regions by eyes opening and over the central parietal regions by movement and sensory stimulation. The occurrence of dynamic changes in spontaneous electroencephalogram (EEG) rhythms in the awake state or sleep is highly variable. These rhythms can be externally modulated with a perturbation method to trigger oscillatory brain activity. The method involves delivering an external stimulus by transcranial magnetic stimulation (TMS) and recording its effects on cortical activity by EEG. Advanced EEG systems compatible with TMS (EEG-TMS co-registration) offer the ability to study EEG reactivity in humans in the awake state.

Most studies to date have focused on slow EEG responses evoked by a single magnetic stimulus in the time domain (Ilmoniemi et al., 1997; Izumi et al., 1997; Paus et al., 1998, 2001; Komssi et al., 2002; Thut et al., 2003; Bonato et al., 2006; Thut and Pascual-Leone, 2010; Del Felice et al., 2011) by investigating more complex and widespread brain oscillatory activity induced by external stimulation (Thut and Miniussi, 2009). The application of EEG-TMS co-registration to high frequencies instead of low frequencies (i.e., cortical evoked potentials) has opened new and intriguing lines of research, yielding a wealth of data on rhythmic brain activities (Fuggetta et al., 2008; Thut and Miniussi, 2009; Manganotti et al., 2012) and on the connectivity of brain areas during the wake state or sleep (Massimini et al., 2005, 2007, 2009). Indeed, single-pulse TMS (Paus et al., 2001; Fuggetta et al., 2005; Van Der Werf et al., 2006; Rosanova et al., 2009; Manganotti et al., 2012) or a TMS pulse train (Brignani et al., 2008; Fuggetta et al., 2008; Plewnia et al., 2008; Noh et al., 2012) induce synchronous rhythmic rapid brain activity that preferably oscillates in the natural frequency of the target site. Such an experimental paradigm was proposed by Johnson et al. as a way to clarify the behavioral effects of TMS, e.g., by studying TMS-induced oscillatory activity modifications (Johnson et al., 2010).

Although the real meaning and site of rapid oscillatory synchronization evoked by TMS remain to be elucidated, cortical and subcortical sources have been suggested (Van Der Werf et al., 2006; Rosanova et al., 2009). In a previous work (Manganotti et al., 2012), we documented the different and dynamic time course of all frequencies, defined as slow (delta and theta) and fast (alpha and beta) activities, after single, paired and transcallosal TMS using wavelet time-frequency analysis, where we suggested possible inhibitory network activation by brain stimulation in the rest awake state for these synchronized evoked rhythms. This method is appealing for studying different states of the brain and it is feasible with different EEG systems.

Recent research into the effects of sleep and sleep deprivation has largely focused on standard TMS parameters, so-called transcranial evoked potentials (TEPs), which are the slow, early components recorded on EEG after a TMS pulse. Standard TMS studies have shown decreased motor excitability in normal subjects during sleep (Manganotti et al., 2001; Grosse et al., 2002; Avesani et al., 2008), while a discordant effect on motor evoked potentials (MEPs), with a mild amplitude decrease according to Manganotti et al. (2001) or an increase according to Civardi et al. (2001), was described in normal subjects after sleep deprivation. Conversely, sleep deprivation in epileptic patients results in a marked increase in cortical excitability (Manganotti et al., 2006). TEP modulation by vigilance states appears to be more nuanced. While the reproducible slow components evoked by TMS during wake and sleep have been identified (Komssi et al., 2004; Bonato et al., 2006; Massimini et al., 2007), the main difference with standard TMS parameters lies in the marked increase in the amplitude of evoked potentials during NREM sleep (Massimini et al., 2009; Del Felice et al., 2011) and during anesthesia (Ferrarelli et al., 2010), with a pronounced increase seen after sleep deprivation (Del Felice et al., 2011). Indeed, Huber et al. (2013) observed that the excitability of the human frontal cortex, measured as the immediate (0–20 ms) EEG reaction to TMS, progressively increases with time awake, from morning to evening and after one night of total sleep deprivation, and that it decreases after recovery sleep. Finally, in an altered hyperexcitable cortex, as in epilepsy, TEPs reflect this state, showing an impressive augmentation in amplitude during sleep and particularly after sleep deprivation (Del Felice et al., 2011).

The aim of this study was to investigate slow and fast oscillatory activities synchronized by single-pulse TMS delivered over the primary motor area (M1) in the time-frequency domain during wake, NREM sleep, and sleep deprivation. The time-frequency approach was applied to detect dynamic changes in the regional neural oscillatory activity of cortical areas and to relate the modulation of these induced oscillations to the different brain states.

## MATERIALS AND METHODS

### SUBJECTS

The study sample was 6 healthy subjects (3 men and 3 women; mean age, 28.6 years ± standard deviation 4.7 years), right-handed as assessed by the Edinburgh Handedness Inventory (Oldfield, 1971). None of the subjects had a medical history of neurological disease or was taking any medications. Basal EEG was normal in all subjects. Sleep was scored according to American Academy of Sleep Medicine (AASM) guidelines on monopolar montage, considering frontal, central and occipital leads, with additional electro-oculogram and electromyogram derivations on a 30 s basis (Silber et al., 2007). Continuous EEG recordings showed unmistakable N1 and N2 sleep stages in all subjects. Only brief lapses of N3 sleep stage were scored in the majority subjects. None of the EEG recordings showed REM sleep (see **Table 1** for polysomnographic data). All subjects initially experienced difficulty in falling asleep owing to the effect of TMS before entering a distinct sleep stage. In accordance with the Declaration of Helsinki, written informed consent to participate in the study was obtained. The study design and protocol were approved by the Local Ethics Committee of the Verona University Department and Hospital.

**Table 1:** Polysomnographic parameters. Mean and standard deviation.

PSG parameters	
Total sleep time	53.4 (±10.7) min
Total recording time	103 (±18.8) min
Sleep onset latency	27.2 (±10.8) min
WASO	12.4 (±9.6) min
NREM	100%
REM	0%
N1	37 (±4.1) min
N2	49 (±6.5) min
N3	14 (±3.2) min

*WASO, wakefulness after sleep onset, NREM, non-rapid eye movement, REM, rapid eye movement, N1, N2, N3, NREM sleep stages.*

### EEG RECORDINGS

Electroencephalogram data were acquired using a magnetic resonance (MR)-compatible EEG amplifier (BrainAmp 32MRplus, BrainProducts GmbH, Munich, Germany) and a cap providing 30 TMS-compatible coated-electrodes positioned according to a 10/20 system. Additional electrodes were used as ground (AFz) and reference (FCz). The EEG data were bandpass-filtered at 0.1–500 Hz and digitized at a sampling rate of 5 KHz.

### TMS STIMULATION

Transcranial magnetic stimulation was performed using a Magstim-Rapid Stimulator in biphasic pulse configuration (Magstim Company Ltd, London, UK) which generates a maximum magnetic field of 1.5 T. TMS was delivered through a figure-of-eight focal coil oriented so that the induced electric current flowed in a posterior-anterior direction over the left M1. MEPs were recorded from the right thenar eminence (TE) muscle with Ag/AgCl surface electrodes fixed to the skin with a belly tendon montage. The coil was placed tangentially to the scalp, with the handle pointing backwards and laterally at a 45° angle away from the midline. The stimulation coil was positioned with the handle pointing backwards and over the optimal scalp position to obtain the highest MEP, corresponding approximately to between C3 and P3 in all subjects. Induced currents were directed postero-anteriorly. Stimulus intensity was set at 110% of motor threshold (MT) intensity. MT intensity was approached from individual suprathreshold levels by reducing the stimulus intensity in 1% steps. MT intensity was defined as the lowest stimulator output intensity capable of inducing MEPs of at least 50 μV peak-to-peak amplitude in relaxed right TE muscles in at least half of 10 trials over the optimal scalp position (Rossini et al., 1994). Stimulus intensities are expressed as a percentage of maximum stimulator output.

The click associated with the coil discharge propagates through air and bone and can elicit an auditory N1–P2 complex at latencies of 100–200 ms (Nikouline et al., 1999; Tiitinen et al., 1999). In this study, we inserted earphones to mask the coil-generated click in all subjects to avoid any effect of clicks in the modulation of cortical oscillatory activities. A white noise (90 dB) was played through the inserted earphones to mask the coil-generated click (Fuggetta et al., 2005). All subjects confirmed that the white noise was sufficient to mask the auditory input.

### EXPERIMENTAL DESIGN

The subjects were asked to maintain a regular sleep schedule for at least 5 days prior to the beginning of the study. The first TMS recording was performed between 1 p.m. and 3 p.m. in basal conditions (T_0_), and the second TMS recording from 1 p.m. to 3 p.m. the day after partial sleep deprivation (T_1_). Partial sleep deprivation was achieved by having the subject stay awake from 3 a.m. until morning; no napping was permitted. Sleep was recorded in the same session as the sleep deprivation recording, immediately after the latter (T_2_). Subjects were asked to refrain from taking any stimulating substances (e.g., coffee, cola, smoking). The EEG recording was performed before, during and after each TMS stimulation. An EEG baseline acquisition in resting state condition was also performed before the beginning of each stimulation (before T_0_, T_1_, and T_2_), with eyes open at T_0_ and T_1_ and with eyes closed at T_2_. Although the eyes open condition produced a higher number of trials that had to be discarded due to blinking artifacts, it ensured the subjects did not fall asleep during the experiment. During the awake and sleep deprivation recordings, the subjects were seated in an armchair with the elbow semi-flexed; the forearm was pronated, fully relaxed and supported by the arm of the chair. During the sleep recordings, the subjects lay in bed in a dark, sound proof laboratory room; the head was reclined over an *ad hoc* tailored foam-rubber pillow to allow correct positioning of the coil over the scalp (Del Felice et al., 2011). To ensure that masking would be effective, the subjects had the earphones inserted with the white noise turned on for the entire duration of the experiment.

**FIGURE 1:**
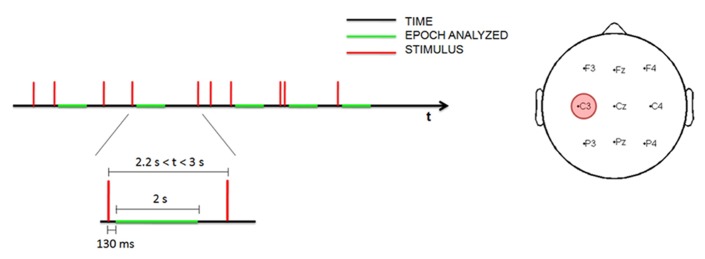
**Time schedule for measuring EEG data during the stimulation (left).** Topographic distribution of the nine electrodes analyzed with the site of stimulation (in red; right).

Transcranial magnetic stimulation single-pulse stimuli were delivered at random, with a minimum inter-trial interval (ITI) of 0.8 s and a maximum of 3 s (**Figure 1**). During the awake and sleep deprivation sessions, at least 150 stimuli were administered; during the sleep session, stimulation continued throughout the entire sleep period.

### WAVELET ANALYSIS

The EEG data were analyzed using a time-frequency procedure to characterize TMS-induced oscillations (Manganotti et al., 2012). The EEG data were downsampled to 250 Hz and all recordings were visually inspected; trials with artifacts produced by environmental noise, muscle activity or eye movement were rejected. Only trials recorded during N1–N2 stages were included in the analysis, whereas trials recorded during N3 stage were excluded in order to ensure the maximum possible homogeneity. Epochs with interstimulus interval greater than 2.2 s were selected for the analysis, to ensure the results were comparable with the previous study (Manganotti et al., 2012). Since magnetic artifacts were contained in the first 130 ms, the EEG traces were analyzed 130 ms after magnetic stimulation. In this way the early, slow TEP was excluded from the analysis, focusing on the time-course of the power of all frequency ranges during the relative long-term. Trials of 2 s were selected from 130 to 2130 ms after the stimulus by visual inspection.

**FIGURE 2:**
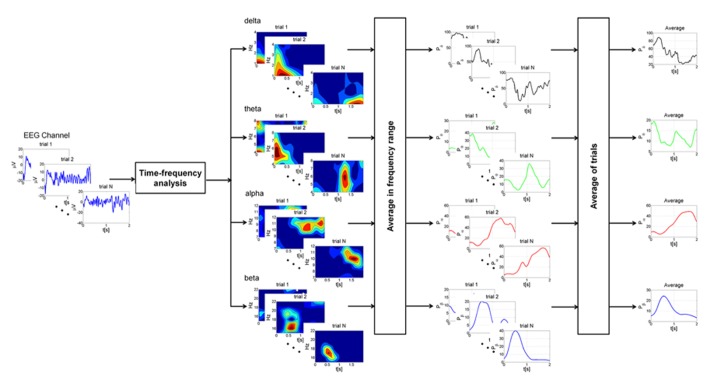
**Schematic representation of the different steps of the analysis at single subject level.** For each EEG channel trials with intervals greater than 2.2 s were selected for the analysis. Time-frequency analysis was performed on each trial with continuous Morlet wavelet transform in the main frequency bands: delta (1–4 Hz), theta (4–8 Hz), alpha (7–12 Hz), and beta (15–22 Hz). The power was then averaged in the frequency ranges of interest, producing a power time course for each trial. Finally, profiles for each trial were averaged and normalized to the baseline value. This procedure was applied for each condition (wake state, sleep deprivation and sleep).

Time-frequency analysis was performed on the most representative channels (F3, Fz, F4, C3, Cz, C4, P3, Pz, and P4) with continuous Morlet wavelet transform, which provides a time course after magnetic stimulation of the relative power in the main frequency bands: delta (1–4 Hz), theta (4–8 Hz), alpha (7–12 Hz), and beta (15–22 Hz). A family of Morlet wavelets was constructed at 1 Hz frequency intervals ranging from 1 to 30 Hz. Each wavelet function has a Gaussian distribution in the time (SD: σ_t_) and frequency domains (SD: σ_f_) around the center frequency *f*_0_ and it depends on a parameter, the number of oscillations (*f*_0_/σ_f_), which has to be chosen by the user. The number of oscillations in each data window can be critical. There is no rule for determining this parameter. After several attempts, we choose these parameters because we could best investigate power changes as the optimal compromise in time-frequency using a 1–30 Hz frequency range and a temporal window of 2 s. Our wavelet family was computed using a ratio of 4 oscillations for delta, 8 for theta, 12 for alpha and 22 for beta bands (coinciding with the highest frequency). 20 epochs of 2 s of basal EEG devoid of artifacts were selected for each subject and for each condition. The reference baseline spectra was calculated by averaging wavelet spectra across time and frequency, obtaining one value for each band. The mean and the standard deviation of relative power for each channel were computed. Profiles for each subject were averaged from the post-stimulus trials (a mean of 86 epochs of 2 s at T_0_, 94 at T_1_ and 455 at T_2_; **Figure 2**) and normalized to the baseline value (expressed as 1) after the grand-average. The EEG baseline acquisition was performed in a resting state condition, differently from TEP study paradigms, where the evoked high amplitude EEG deflections are compared to a very short epoch of some ms preceding the stimulus. Nevertheless, our aim was to compare changes of oscillatory activity induced on EEG by a series of stimulation during the three vigilance states to a reference value, devoid of any pre-planned external perturbation, in a 2 s interval, thus evaluating the long term effect of the stimulation and not the early short-lasting TEP.

ANOVA for repeated measures was applied to relative powers with the factors “condition” (T_0_, T_1_, T_2_) and “time point” (number of time point in 2 s: 500). *Post-hoc* paired *t*-test adjusted for multiple comparisons with Bonferroni method was used. Statistical significance was set at *p* < 0.05. In order to check whether post-stimulus activity differed significantly from the basal level, a paired samples *t*-test was performed at each sampling time (*p* < 0.05) to evidence the intervals during which the relative power differed significantly from baseline.

## RESULTS

The times/latencies below mentioned, in which EEG modifications were observed, refer to the time in the processed epochs and not latency with respect to TMS. Trials of 2 s were indeed selected from 130 to 2130 ms after the stimulus.

### ALPHA BAND

**FIGURE 3:**
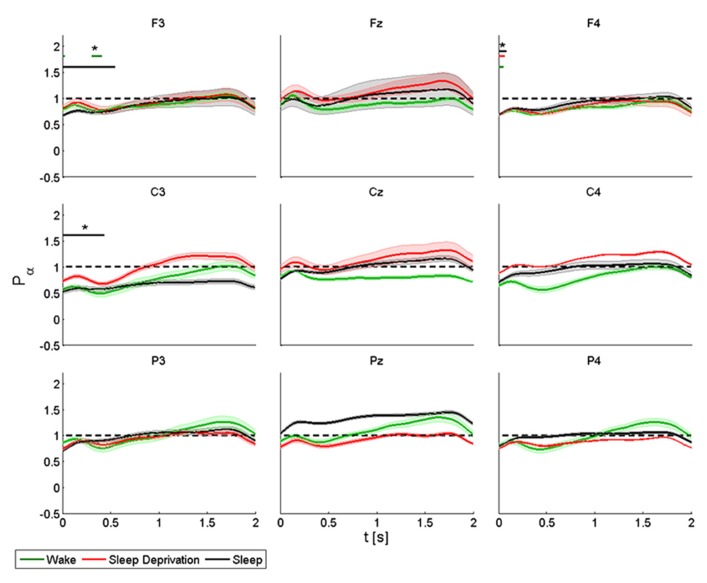
**Average (*N* = 6) relative wavelet power and standard error in alpha range (7–12 Hz), after single-pulse TMS, during wake (green), sleep deprivation (red), and sleep (black).** Asterisk (*) above the bars indicates values significantly different from basal level. Data are analyzed from 130 to 2130 ms after the stimulus onset.

In the alpha band, TMS induced a decrease of power in proximity of the stimulation site, followed by progressive synchronization in time, especially over the frontal and central electrodes. Basal conditions returned about 1 s or more after the stimulation (**Figure 3**).

The power of the *frontal electrodes* (F3-Fz-F4) decreased (by about 20%) in the 1-s period during the wake state and sleep deprivation (time limited significant decrease in F4 at T_0_, T_1_, and T_2_), followed by a more evident increase lasting from 1 to 2 s; the decrease was less clear over Fz. During sleep, the power decrease was more evident (significant decrease in F3) (40% in F3 and F4). An asterisk above the bars indicates a statistically significant difference between the post-stimulus activity and the basal value. A similar pattern was observed over the *central electrodes* (C3-Cz-C4): during the wake state and after sleep deprivation, the power decreased (by 50% at T_0_ and 30% at T_1_in C3) over baseline during the first 1-s period, followed by an increase lasting from 1 to 2 s, which was clearly visible over C3. The power during sleep decreased significantly by about 30% only in C3. This pattern, though also observable over the *parietal electrodes* (P3-Pz-P4), was not significant.

ANOVA testing the alpha power for each electrode disclosed a significant main effect for the factor “condition” (T_0_, T_1_, and T_2_) in Fz (*F*(2,10) = 4.192, *p* < 0.05). No significant differences were observed between the conditions.

### BETA BAND

**FIGURE 4:**
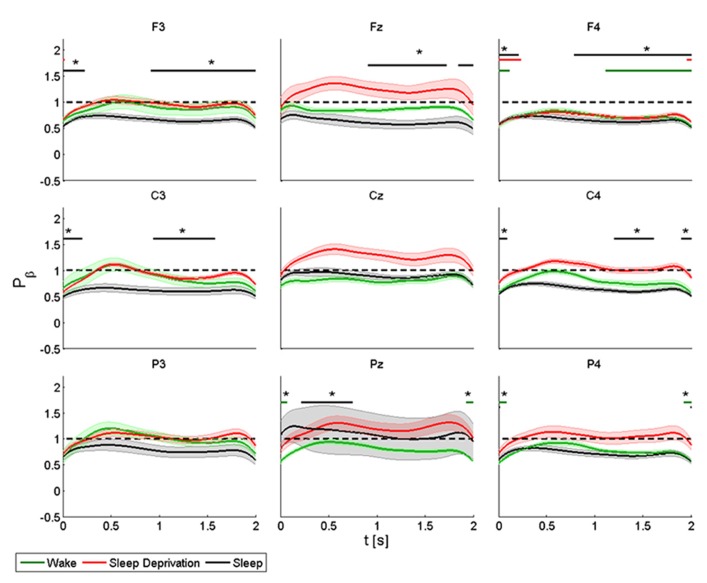
**Average (*N* = 6) relative wavelet power and standard error in beta range (15–22 Hz), after single-pulse TMS, during wake (green), sleep deprivation (red), and sleep (black).** Asterisk (*) above the bars indicates values significantly different from basal level. Data are analyzed from 130 to 2130 ms after the stimulus onset.

We observed a more rapid initial decrease in power in the beta band than in the alpha band, followed by an increase in power again more marked in proximity of the stimulation site and the homolateral frontal regions, but also over the parietal areas (**Figure 4**).

The power of the *frontal electrodes* (F3-Fz-F4) decreased significantly in F3 and F4 by about 40% after sleep and by more than 40% during all three conditions in F4. The power over the F4 electrode remained below the baseline value during the entire post-stimulus interval. An increase lasting from 1 to 2 s was observed in the three frontal electrodes during all the conditions. During sleep, the power of the *central electrodes* (C3-Cz-C4) decreased (significant decrease of 50% in C3 and C4) from the baseline value and lasted 0.3 s, followed by an evident rebound. In Cz the power at T_0_ and T_2_ remained below the baseline value during the entire post-stimulus interval, even if no significant change was noted. After stimulation, the power of the *parietal electrodes* (P3-Pz-P4) decreased significantly from the baseline value in Pz and in P4 at T_1_. A significant power modification from the baseline was also observed in Pz during sleep from 0.2 to 0.7 s.

ANOVA testing the beta power for each electrode disclosed a significant main effect for the factor “condition” (T_0_, T_1_, and T_2_) only in Cz (*F*(2,10) = 6.778, *p* < 0.05). No significant differences were observed between conditions.

### THETA BAND

**FIGURE 5:**
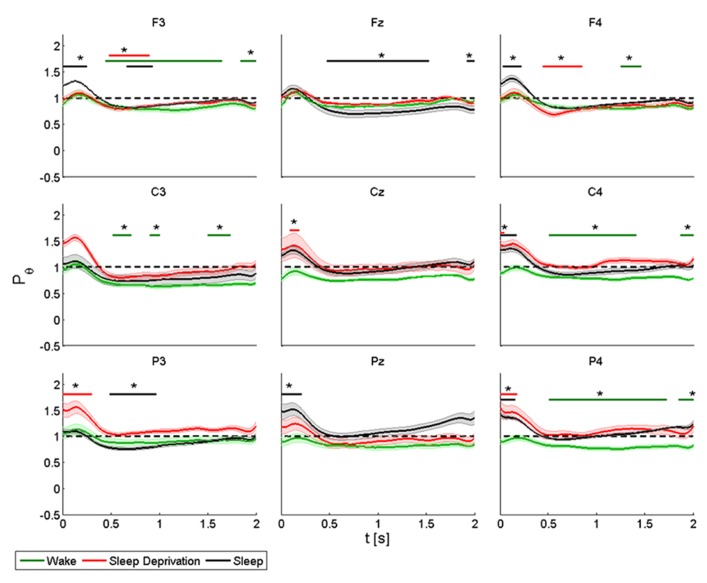
**Average (*N* = 6) relative wavelet power and standard error in theta range (4–8 Hz), after single-pulse TMS, during wake (green), sleep deprivation (red), and sleep (black).** Asterisk (*) above the bars indicates values significantly different from basal level. Data are analyzed from 130 to 2130 ms after the stimulus onset.

An increase in amplitude of theta relative power was observed in the first 0.3 s (**Figure 5**).

During the wake state and after sleep deprivation, the power of the *frontal electrodes* (F3-Fz-F4) increased markedly but not significantly from the baseline value, with a maximum at 0.2 s and lasting 0.5 s; while the power increased significantly during sleep in F3 and F4. A significant decrease was also observed in F3 from 0.5 to 2 s at T_0_, from 0.5 to 1 at T_1_ and from 0.7 to 1 s at T_2_; in Fz from 0.5 to 1.5 s at T_2_; in F4 from 0.5 to 1 s at T_2_ and from 1.3 to 1.5 s at T_1_. A similar pattern was observed in the *central electrodes* (C3-Cz-C4), but the increase was significant only after sleep deprivation in Cz and C4 and during sleep in C4. During the wake state, the power remained below the baseline value for the entire interval of 2 s, and this trend was significant for C3 and C4. The same pattern was also observed in the *parietal electrodes* (P3-Pz-P4), but the power increased significantly (by about 40%) only in P3 and in P4 during sleep deprivation and in Pz and in P4 during sleep. The decrease in power after 0.5 s was significant in P3 during sleep, and in P4 during the wake state.

ANOVA testing the theta power for each electrode disclosed a significant main effect for the factor “condition” (T_0_, T_1_ and T_2_) in P4 (*F*(2,10) = 3.956, *p* < 0.05). A *t*-test disclosed a significant difference between wake state and sleep (*p* < 0.05) from 0 to 0.08 s and between sleep deprivation and sleep (*p* < 0.05) from 1.6 to 2 s.

### DELTA BAND

**FIGURE 6:**
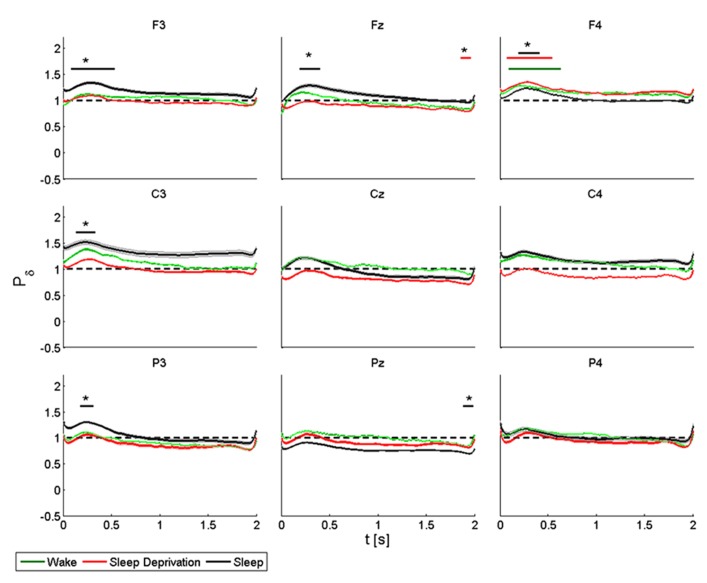
**Average (*N* = 6) relative wavelet power and standard error in delta range (1–4 Hz), after single-pulse TMS, during wake (green), sleep deprivation (red), and sleep (black).** Asterisk (*) above the bars indicates values significantly different from basal level. Data are analyzed from 130 to 2130 ms after the stimulus onset.

The delta rhythm was characterized by synchronization, with a peak at about 0.3 s, followed by a gradual reduction in power until returning to the basal condition (**Figure 6**).

During the wake state and after sleep deprivation, the power of the *frontal electrodes* (F3-Fz-F4) increased significantly by about 40% in F4 over the baseline value, with a maximum at 0.3 s and lasting 1 s. This pattern was also observed in F3 and Fz. During sleep, the power increased significantly in F3, Fz and F4, peaking after 0.3 s. In Fz a significant difference from the baseline value was observed from 1.8 to 2 s during T_1_. During T_0_, T_1_ and T_2_, the power of the *central electrodes* (C3-Cz-C4) increased from the baseline value, with a maximum at 0.3 s and lasting less than 1 s. There was a significant increase only in C3 during sleep. After sleep deprivation, the power remained below the baseline value in Cz and C4. The pattern, though also observable in the *parietal electrodes* (P3-Pz-P4), was significant only in P3, where the power increased from the baseline with a maximum at 0.3 s during sleep, and in Pz where the power remained below the baseline from 1.8 to 2 s during sleep.

ANOVA testing the delta power for each electrode disclosed a significant main effect for the factor “condition” (T_0_, T_1_, and T_2_) in F3 (*F*(2,10) = 34.626, *p* < 0.05) and in C4 (*F*(2,10) = 3.849, *p* < 0.05). A *t*-test disclosed a significant difference only in F3 between wake state and sleep from 0.8 to 1.2 s (*p* < 0.05).

## DISCUSSION

This study investigated the time course of different patterns of the main brain oscillatory activities after brain stimulation during different states of vigilance: wake, sleep deprivation and sleep. Insight on externally modulated brain rhythm patterns can be gained from the effects of single-pulse TMS on brain oscillations. Single-pulse TMS can induce synchronization of rapid brain rhythms (Paus et al., 2001; Fuggetta et al., 2005; Van Der Werf et al., 2006; Rosanova et al., 2009; Manganotti et al., 2012), and the behavioral correlates of such oscillatory modulations have been identified in the phase locking of ongoing brain oscillations (Dugué et al., 2011), the encoding of different aspects of the stimulus by different frequencies (“multiplexing;” Siegel et al., 2009; Panzeri et al., 2010), or by aligning two oscillatory neural populations to their high excitability phase (“communication-through-coherence” Fries, 2005). The novelty of the present method stands in the application of a time-frequency analysis to the time course of different rapid and slow brain oscillations induced by TMS not only in an awake state, but also in sleep deprivation and sleep.

The dynamic and short-time modulation of brain activity is confirmed by the fact that low-intensity single-pulse TMS, applied on the sensorimotor areas in the awake state, induces early desynchronization over the frontal and central-parietal electrodes, followed by a rebound of synchronization in the alpha and beta bands, paralleled by early synchronization in delta and theta activities and subsequent desynchronization. This pattern reveals the distinct behavior of oscillations after an external perturbation, (TMS), and suggests the possibility to differentiate each brain rhythm solely on the basis of a single shock of depolarization by TMS (Manganotti et al., 2012). In the awake state, this pattern of EEG reactivity to TMS was consistent in our group of subjects.

The main study finding was that this pattern can be also slightly affected by brain intrinsic states: during light NREM sleep (N1 and N2) there was slight precocious desynchronization of alpha over the stimulated areas, with a minimal contralateral effect, as well as a similar desynchronization of beta band rhythms over the antero-central cortex, whereas an early, not well localized increase in theta synchronization, and a more anterior and site-related synchronization of delta emerged again in sleep. Given that the stimulation site was over the primary motor area, this peculiar localization of rhythms responses bears an important physiological meaning that will be discussed below. Indeed, these results are of interest also because they seem to be maintained throughout the trials, where a potential bias could have been generated by the very short ITI intervals. The very short ITI might influence the findings, since we reported results obtained analyzing epochs with ITI greater than 2.2 s, discarding the epochs shorter. Differences found in oscillatory activity could possibly not completely depend on stimulation itself, but also to some spurious state-dependent differences between the two EEG states (resting state/baseline condition and stimulation conditions). Indeed, the reproducibility of the results throughout states and subjects hints to the major effect of TMS, that could nonetheless be endowed with endogenous processes, the course of which were beyond the aim of our study. Since not all stimulations were delivered in exactly the same condition, the persistence of analogous responses adds robustness to our data and possibly points to a strong modulating effect of vigilance states, in which the neurotransmitters balance is distinct in each condition, on brain rhythms. Interestingly, partial sleep deprivation was not responsible for any clear-cut modulating effect, except for a minor desynchronization of theta over the frontal bilateral leads and a precocious synchronization over the posterior areas, in contrast with our previous data (Del Felice et al., 2011) on the massive impact of sleep deprivation on cortical excitability.

### WAKE OSCILLATORY ACTIVITY

Early research on EEG-TMS co-registration reported only synchronization in beta activity after single magnetic stimulation and linked it to a sort of resetting or disruption of the ongoing oscillatory activity of M1 produced by external magnetic stimulation of the brain (Paus et al., 2001). Fuggetta et al. observed that single-pulse TMS produces an increase in power in both the beta and alpha bands, unlike the self motor finger movement which produces a well-known decrease in alpha and beta powers. Also, using MT and minimal intensity stimulation, they noted a significant effect on brain modifications, which suggests an effect of magnetic stimulation on the cortical sources (Fuggetta et al., 2005).

An increase in beta power has been observed by other authors (Van Der Werf et al., 2006; Rosanova et al., 2009). However, in most of these studies, spectral estimation was performed using fast Fourier transform which does not detect dynamic changes. To overcome this limitation, methods that can monitor the temporal variation of EEG power are needed. In this study we used a wavelet-based method to detect the temporal modulation of brain oscillations in the main frequency bands. This approach has already been applied to experiments with single, paired-pulse and transcallosal TMS (Manganotti et al., 2012). Single pulse, paired-pulse and transcallosal TMS, which investigate intra- and transcortical inhibition, induce similar patterns. Specifically, single pulse TMS provokes an initial mainly anterior decrease of power in the alpha and beta bands, followed by a more prominent increase of power in beta activity over the ipsilateral and contralateral M1. Similar results are observed also on our data, although the significance is not robust as in our previous study, with a minimal precocious desynchronization of the anterior alpha and a slow desynchronization of beta mainly over the contralateral motor area, as well as the late desynchronization of theta over the frontal and central areas, mainly on the stimulated site. These results, in line with the previous ones (Manganotti et al., 2012), differ from other literature data on the basis of the innovative analysis approach that defines the time course of the oscillation and does not rely on an averaged analysis.

Finally, the spontaneous EEG signal is the indistinguishable summation of the activation of both fast and slow excitatory postsynaptic potentials (fEPSPs and sEPSPs, respectively) as well as fast and slow inhibitory postsynaptic potentials (fIPSPs and sIPSPs, respectively) (Rosenthal et al., 1967). fIPSPs are mediated by γ-aminobutyric acid (GABA_A_) postsynaptic receptors lasting approximately 20–30 ms (Davies et al., 1990). TMS can explore the inhibitory system of motor areas. Short intracortical inhibition ([SICI] as evaluated by means of MEP amplitude modulation at ISI 3 ms) is thought to explore the net effect of the activation of inhibitory GABA_A_ circuits in M1. We can hypothetically exclude that single TMS, which induces patterns similar to paired TMS on brain oscillations, can investigate similar inhibitory circuits in sleep and sleep deprivation.

### SLEEP DEPRIVATION, BRAIN EXCITABILITY AND OSCILLATORY RHYTHMS

The homeostatic process regulates the propensity to sleep in relation to the length of prior wakefulness (Borbély and Achermann, 1999), and the amount slow-wave sleep (SWS) during subsequent NREM sleep has been proposed as a measurement for sleep homeostasis, equated to sleep intensity or sleep depth (Steriade, 2005). Sleep deprivation impairs cognition and performance, as measured on verbal and non-verbal memory tasks (Smith and MacNeill, 1994; Walker et al., 2002a,b), and cognitive and attentional abilities (Linde and Bergstrom, 1992; Harrison and Horne, 1999; Doran et al., 2001; Belenky et al., 2003; Drummond et al., 2006; Kendall et al., 2006), and primarily affects frontal executive functions. In clinical practice, sleep deprivation is an established method to provoke EEG epileptiform abnormalities (Bennett, 1963; Pratt et al., 1968; Jovanovic, 1991; King et al., 1998) and seizures in most types of epilepsy (Dinner, 2002). Neurophysiologically, sleep deprivation modulates the frequency power of EEG rhythms, enhancing the frontal predominance of delta (Kattler et al., 1994; Achermann et al., 2001), with a main effect over the left hemisphere, increasing theta rhythms (Cajochen et al., 1995; Dumont et al., 1999) predominantly over the frontal (Cajochen et al., 1995) and temporal sites (Forest and Godbout, 2000), and increasing alpha in an eyes-open condition (Stampi et al., 1995; Corsi-Cabrera et al., 1996) while decreasing it in an eyes-closed state (Drapeau and Carrier, 2004).

A brain stimulation paradigm has rarely been applied to investigate sleep deprivation. A recent study by Del Felice et al. (2011) looked into its effects on TEPs in a group of healthy volunteers and patients with generalized epilepsy (juvenile myoclonic epilepsy, JME). The authors found that sleep deprivation enhanced cortical excitability, as measured by TEP amplitude, more markedly over the frontal areas in the epileptics, possibly due to involvement of the frontal cortex in the pathogenesis of JME. EEG-TMS was also used to monitor cortical excitability in healthy individuals as a function of time awake (Huber et al., 2013). The authors observed that the immediate cortical response to direct stimulation progressively increases with time awake.

Our data detected a possible theta synchronizing effect of sleep deprivation over the posterior cortical areas, whereas a not significant phenomenon was observed over the frontal electrodes. The almost negligible effect that sleep deprivation exerts on late-response brain oscillation contrasts with the strong effect it demonstrates on TEP. Although we do not have an explanation for this observation, we could suppose a different susceptibility to sleep deprivation effects of the superficial TEPs generators that rely on short interconnections in contrast to the long-loop, deep structures that reverberate in the long-term aftermath of the TMS.

Nonetheless, the reaction we were able to demonstrate seems to bear a physiological meaning. Since theta rhythm is the signature of sleepiness – the so called theta of drowsiness – the response to an external perturbation of a sleepy brain appears to reflect its physiological state: a disturbed brain increments these oscillations in the frequency range more abundant in the stage it is in and over the areas where it is usually represented – i.e., theta over the temporal and parietal. An experimental paradigm aiming at simulating a higher sleep pressure proved an analogous brain reaction: Kirov et al. (2009) applied transcranial slow oscillation stimulation at a frequency of 0.75 Hz during the wake state observing a diffuse theta increase. Although in their experiment a rhythmic entraining effect might have artificially boosted slow brain oscillations, in our view a physiological state of increased slowed rhythms, such as sleep deprivation, has the intrinsic capacity to reverberate at the frequency that is more consistent with its state – theta rhythm in the specific case.

### SLEEP AND OSCILLATORY RHYTHMS

No general consensus exists on the generation of ongoing EEG rhythms in wake and in sleep. The prevailing model according to Steriade sees corticofugal slow oscillations (<1 Hz) that group thalamic-generated delta rhythms (1–4 Hz) and spindling activity (7–14 Hz), with delta dominating the EEG and low amplitude alpha (8–12 Hz; Steriade, 2003). When arousing stimuli, either exogenous or endogenous, are delivered, spindling, slow and ultraslow oscillations are blocked by inhibition of the reticulothalamic (7–14 Hz), thalamocortical (1–4 Hz) and intracortical (<1 Hz) generators. They are replaced by beta (12–18 Hz) and gamma (up to 40 Hz) rhythms paced by the basal forebrain (Steriade, 2003). Instead, during the wake state, alpha is the dominant rhythm, with a low amplitude delta (Steriade and Llinás, 1988; Pfurtscheller and Lopes da Silva, 1999), suggesting a reciprocal inhibition between their generators.

Our data seem to provide the experimental setting to replicate the aforementioned rhythms alternation: during sleep, the brain response to TMS consisted of an early alpha desynchronization over the stimulation site, with a minimal effect over the contralateral omologous areas (possibly due to the transcallosal spread) coupled with an early delta synchronization over the anterior areas and over the stimulation site. Of interest is the main anterior effect on the slow rhythm: the frontal cortex is the main source of the so-called slow traveling wave of sleep (Massimini et al., 2004; Ferri et al., 2005; Murphy et al., 2009) over which there is a higher chance of eliciting, through TMS pulses, waves resembling the physiological oscillation of SWS (Massimini et al., 2004). This mechanism, associated with a recently demonstrated entrainment phenomenon, could contribute to the observed higher delta power over the frontal brain area. As demonstrated by Veniero et al. (2011), stimulating at the frequency of the underlying brain rhythms (alpha in that case) leads to a progressively enhanced oscillatory response in the same frequency band. In our experiment, this seems to hold true over the anterior areas, where the slow wave is physiologically generated, and over the stimulated hemisphere, which is thus the more solicited.

To sum up, our results hint to the change of tagging the brain physiological state by its reaction to external perturbations: the coupled desynchronization of faster, wake-like rhythms, and the rise of slow ones induced by an external perturbation during sleep could indicate that the brain reacts accordingly to its actual state.

### CONCLUSION

Our results show a reciprocal synchronizing/desynchronizing effect on slow and fast oscillatory activity in response to focal, standardized TMS after sleep deprivation and sleep, as detected by time-frequency analysis. Nevertheless, this preliminary study has some limitations. Well-designed studies with larger sample size and more detailed data are needed to confirm these conclusions. However, these findings highlight the pronounced interference that external brain stimulation can exert on the cortex modulated by the vigilance state and open up new perspectives regarding automated detection systems of brain state modifications, as well as giving a more detailed insight in the functional modification in pathological conditions. This holds true, for example, in the evaluation of hyperexcitable states, as epilepsy, or states of disturbed consciousness, as in minimal consciousness: the detection of rhythmicity patterns modification could point to a forthcoming clinical manifestation – either a seizure developing or the consciousness level modification, with a potential clinical application.

## Conflict of Interest Statement

The authors declare that the research was conducted in the absence of any commercial or financial relationships that could be construed as a potential conflict of interest.

## References

[B1] Achermann P. Finelli L. A Borbély A. A. (2001). Unihemispheric enhancement of delta power in human frontal sleep EEG by prolonged wakefulness. *Brain Res.* 913 220–223 10.1016/S0006-8993(01)02796-2 11549390

[B2] Avesani M. Formaggio E. Fuggetta G. Fiaschi A. Manganotti P. (2008). Corticospinal excitability in human subjects during nonrapid eye movement sleep: single and paired-pulse transcranial magnetic stimulation study. *Exp. Brain Res.* 187 17–23 10.1007/s00221-008-1274-3 18231786

[B3] Belenky G. Wesensten N. J. Thorne D. R. Thomas M. L. Sing H. C. Redmond D. P. (2003). Patterns of performance degradation and restoration during sleep restriction and subsequent recovery: a sleep dose–response study. *J. Sleep Res.* 12 1–12 10.1046/j.1365-2869.2003.00337.x 12603781

[B4] Bennett D. R. (1963). Sleep deprivation and major motor convulsions. *Neurology* 13 953–958 10.1212/WNL.13.11.953 14079955

[B5] Bonato C. Miniussi C. Rossini P. M. (2006). Transcranial magnetic stimulation and cortical evoked potentials: a TMS/EEG co-registration study. *Clin. Neurophysiol.* 117 1699–1707 10.1016/j.clinph.2006.05.006 16797232

[B6] Borbély A. A. Achermann P. (1999). Sleep homeostasis and models of sleep regulation. *J. Biol. Rhythms* 14 557–568 10643753 10.1177/074873099129000894

[B7] Brignani D. Manganotti P. Rossini P. M. Miniussi C. (2008). Modulation of cortical oscillatory activity during transcranial magnetic stimulation. *Hum. Brain. Mapp.* 29 603–612 10.1002/hbm.20423 17557296 PMC6870908

[B8] Cajochen C. Brunner D. P. Kräuchi K. Graw P. Wirz-Justice A. (1995). Power density in theta/alpha frequencies of the waking EEG progressively increases during sustained wakefulness. *Sleep* 18 890–894 8746397 10.1093/sleep/18.10.890

[B9] Civardi C. Boccagni C. Vicentini R. Bolamperti L. Tarletti R. Varrasi C. (2001). Cortical excitability and sleep deprivation: a transcranial magnetic stimulation study. *J. Neurol. Neurosurg. Psychiatry* 71 809–812 10.1136/jnnp.71.6.809 11723210 PMC1737655

[B10] Corsi-Cabrera M. Arce C. Ramos J. Lorenzo I. Guevara M. A. (1996). Time course of reaction time and EEG while performing a vigilance task during total sleep deprivation. *Sleep* 19 563–569 8899935 10.1093/sleep/19.7.563

[B11] Davies C. H. Davies S. N. Collingridge G. L. (1990). Paired-pulse depression of monosynaptic GABA-mediated inhibitory postsynaptic responses in rat hippocampus. *J. Physiol.* 424 513–531 2167975 10.1113/jphysiol.1990.sp018080 PMC1189826

[B12] Del Felice A. Fiaschi A. Bongiovanni G. L. Savazzi S. Manganotti P. (2011). The sleep-deprived brain in normals and patients with juvenile myoclonic epilepsy: a perturbational approach to measuring cortical reactivity. *Epilepsy Res.* 96 123–131 10.1016/j.eplepsyres.2011.05.015 21726980

[B13] Dinner D. S. (2002). Effect of sleep on epilepsy. *J. Clin. Neurophysiol.* 19 504–513 10.1097/00004691-200212000-00003 12488781

[B14] Doran S. M. Van Dongen H. P. Dinges D. F. (2001). Sustained attention performance during sleep deprivation: evidence of state instability. *Arch. Ital. Biol.* 139 253–267 11330205

[B15] Drapeau C. Carrier J. (2004). Fluctuation of waking electroencephalogram and subjective alertness during a 25-hour sleep-deprivation episode in young and middle-aged subjects. *Sleep* 27 55–60 14998238 10.1093/sleep/27.1.55

[B16] Drummond S. P. Paulus M. P. Tapert S. F. (2006). Effects of two nights sleep deprivation and two nights recovery sleep on response inhibition. *J. Sleep Res.* 15 261–265 10.1111/j.1365-2869.2006.00535.x 16911028

[B17] Dugué L. Marque P. VanRullen R. (2011). The phase of ongoing oscillations mediates the causal relation between brain excitation and visual perception. *J. Neurosci.* 31 11889–11893 10.1523/JNEUROSCI.1161-11.2011 21849549 PMC6623205

[B18] Dumont M. Macchi M. M. Carrier J. Lafrance C Hébert M. (1999). Time course of narrow frequency bands in the waking EEG during sleep deprivation. *Neuroreport* 10 403–407 10.1097/00001756-199902050-00035 10203343

[B19] Ferrarelli F. Massimini M. Sarasso S. Casali A. Riedner B. A. Angelini G. (2010). Breakdown in cortical effective connectivity during midazolam-induced loss of consciousness. *Proc. Natl. Acad. Sci. U.S.A.* 107 2681–2686 10.1073/pnas.0913008107 20133802 PMC2823915

[B20] Ferri R. Rundo F. Bruni O. Terzano M. G. Stam C. J. (2005). Dynamics of the EEG slow-wave synchronization during sleep. *Clin. Neurophysiol.* 116 2783–2795 10.1016/j.clinph.2005.08.013 16253553

[B21] Forest G. Godbout R. (2000). Effects of sleep deprivation on performance and EEG spectral analysis in young adults. *Brain Cogn.* 43 195–200 10857693

[B22] Fries P. (2005). A mechanism for cognitive dynamics: neuronal communication through neuronal coherence. *Trends Cogn. Sci.* 9 474–480 10.1016/j.tics.2005.08.011 16150631

[B23] Fuggetta G. Fiaschi A. Manganotti P. (2005). Modulation of cortical oscillatory activities induced by varying single-pulse transcranial magnetic stimulation intensity over the left primary motor area: a combined EEG and TMS study. *Neuroimage* 27 896–908 10.1016/j.neuroimage.2005.05.013 16054397

[B24] Fuggetta G. Pavone E. F. Fiaschi A. Manganotti P. (2008). Acute modulation of cortical oscillatory activities during short trains of high-frequency repetitive transcranial magnetic stimulation of the human motor cortex: a combined EEG and TMS study. *Hum. Brain. Mapp.* 29 1–13 10.1002/hbm.20371 17318833 PMC6870897

[B25] Grosse P. Khatami R. Salih F. Kühn A. Meyer B. U. (2002). Corticospinal excitability in human sleep as assessed by transcranial magnetic stimulation. *Neurology* 59 1988–1991 10.1212/01.WNL.0000038762.11894.DA 12499500

[B26] Harrison Y. Horne J. A. (1999). One night of sleep loss impairs innovative thinking and flexible decision making. *Organ. Behav. Hum. Decis. Process.* 78 128–145 10.1006/obhd.1999.2827 10329298

[B27] Huber R. Mäki H. Rosanova M. Casarotto S. Canali P. Casali A. G. (2013). Human cortical excitability increases with time awake. *Cereb. Cortex* 23 332–338 10.1093/cercor/bhs014 22314045 PMC3539451

[B28] Ilmoniemi R. J. Virtanen J. Ruohonen J. Karhu J. Aronen H. J. Naatanen R. (1997). Neuronal responses to magnetic stimulation reveal cortical reactivity and connectivity. *Neuroreport* 8 3537–3540 10.1097/00001756-199711100-00024 9427322

[B29] Izumi S. Takase M. Arita M. Masakado Y. Kimura A. Chino N. (1997). Transcranial magnetic stimulation-induced changes in EEG and responses recorded from the scalp of healthy humans. *Electroencephalogr. Clin. Neurophysiol.* 103 319–322 10.1016/S0013-4694(97)00007-2 9277634

[B30] Johnson K. A. Baylis G. C. Powell D. A. Kozel F. A. Miller S. W. George M. S. (2010). Conditioning of transcranial magnetic stimulation: evidence of sensory-induced responding and prepulse inhibition. *Brain Stimul.* 3 78–86 10.1016/j.brs.2009.08.003 20633436

[B31] Jovanovic U. J. (1991). General consideration of sleep and sleep deprivation. *Epilepsy Res. Suppl.* 2 205–215 1760089

[B32] Kattler H. Dijk D. J Borbély A. A. (1994). Effect of unilateral somatosensory stimulation prior to sleep on the sleep EEG in humans. *J. Sleep Res.* 3 159–164 10.1111/j.1365-2869.1994.tb00123.x 10607121

[B33] Kendall A. P. Kautz M. A. Russo M. B. Killgore W. D. (2006). Effects of sleep deprivation on lateral visual attention. *Int. J. Neurosci.* 116 1125–1138 10.1080/00207450500513922 16923682

[B34] King M. A. Newton M. R. Jackson G. D. Fitt G. J. Mitchell L. A. Silvapulle M. J. (1998). Epileptology of the first seizure presentation: a clinical electroencephalographic and magnetic resonance imaging study of 300 consecutive patients. *Lancet* 352 1007–1011 10.1016/S0140-6736(98)03543-0 9759742

[B35] Kirov R. Weiss C. Siebner H. R. Born J. Marshall L. (2009). Slow oscillation electrical brain stimulation during waking promotes EEG theta activity and memory encoding. *Proc. Natl. Acad. Sci. U.S.A.* 106 15460–15465 10.1073/pnas.0904438106 19706399 PMC2730962

[B36] Komssi S. Aronen H. J. Huttunen J. Kesaniemi M. Soinne L. Nikouline V. V. (2002). Ipsi- and contralateral EEG reactions to transcranial magnetic stimulation. *Clin. Neurophysiol.* 113 175–184 10.1016/S1388-2457(01)00721-0 11856623

[B37] Komssi S. Kahkonen S. Ilmoniemi R. J. (2004). The effect of stimulus intensity on brain responses evoked by transcranial magnetic stimulation. *Hum. Brain Mapp.* 21 154–164 10.1002/hbm.10159 14755835 PMC6871924

[B38] Linde L. Bergstrom M. (1992). The effect of one night without sleep on problem-solving and immediate recall. *Psychol. Res.* 54 127–136 10.1007/BF00937141 1620796

[B39] Manganotti P. Bongiovanni L. G. Fuggetta G. Zanette G. Fiaschi A. (2006). Effects of sleep deprivation on cortical excitability in patients affected by juvenile myoclonic epilepsy: a combined transcranial magnetic stimulation and EEG study. *J. Neurol. Neurosurg. Psychiatry* 77 56–60 10.1136/jnnp.2004.041137 16361593 PMC2117394

[B40] Manganotti P. Formaggio E. Storti S. F. De Massari D. Zamboni A. Bertoldo A. (2012). Time-frequency analysis of short-lasting modulation of EEG induced by intracortical and transcallosal paired TMS over motor areas. *J. Neurophysiol.* 107 2475–2484 10.1152/jn.00543.2011 22298825

[B41] Manganotti P. Palermo A. Patuzzo S. Zanette G. Fiaschi A. (2001). Decrease in motor cortical excitability in human subjects after sleep deprivation. *Neurosci. Lett.* 304 153–156 10.1016/S0304-3940(01)01783-9 11343825

[B42] Massimini M. Ferrarelli F. Esser S. K. Riedner B. A. Huber R. Murphy M. (2007). Triggering sleep slow waves by transcranial magnetic stimulation. *Proc. Natl. Acad. Sci. U.S.A.* 104 8496–8501 10.1073/pnas.0702495104 17483481 PMC1895978

[B43] Massimini M. Ferrarelli F. Huber R. Esser S. K. Singh H. Tononi G. (2005). Breakdown of cortical effective connectivity during sleep. *Science* 309 2228–2232 10.1126/science.1117256 16195466

[B44] Massimini M. Huber R. Ferrarelli F. Hill S. Tononi G. (2004). The sleep slow oscillation as a traveling wave. *J. Neurosci.* 24 6862–6870 10.1523/JNEUROSCI.1318-04.2004 15295020 PMC6729597

[B45] Massimini M. Tononi G. Huber R. (2009). Slow waves, synaptic plasticity and information processing: insights from transcranial magnetic stimulation and high-density EEG experiments. *Eur. J. Neurosci.* 29 1761–1770 10.1111/j.1460-9568.2009.06720.x 19473231 PMC2776746

[B46] Murphy M. Riedner B. A. Huber R. Massimini M. Ferrarelli F. Tononi G. (2009). Source modeling sleep slow waves. *Proc. Natl. Acad. Sci. U.S.A.* 106 1608–1613 10.1073/pnas.0807933106 19164756 PMC2635823

[B47] Niedermeyer E. (1999). “The normal EEG of the walking adult,” in *Electroencephalography: Basic Principles, Clinical Applications and Related Fields* eds Niedermeyer E. Lopes da Silva F. (Baltimore: Lippincott Williams and Wilkins) 149–173

[B48] Nikouline V. Ruohonen J. Ilmoniemi R. J. (1999). The role of the coil click in TMS assessed with simultaneous EEG. *Clin. Neurophysiol.* 110 1325–1328 10.1016/S1388-2457(99)00070-X 10454266

[B49] Noh N. A. Fuggetta G. Manganotti P. Fiaschi A. (2012). Long lasting modulation of cortical oscillations after continuous theta burst transcranial magnetic stimulation. *PLoS ONE* 7:e35080 10.1371/journal.pone.0035080 PMC3319628 22496893

[B50] Oldfield R. C. (1971). The assessment and analysis of handedness: the Edinburgh inventory. *Neuropsychologia* 9 97–113 10.1016/0028-3932(71)90067-4 5146491

[B51] Panzeri S. Brunel N. Logothetis N. K. Kayser C. (2010). Sensory neural codes using multiplexed temporal scales. *Trends Neurosci.* 33 111–120 10.1016/j.tins.2009.12.001 20045201

[B52] Paus T. Jech R. Thompson C. J. Comeau R. Peters T. Evans A. C. (1998). Dose-dependent reduction of cerebral blood flow during rapid-rate transcranial magnetic stimulation of the human sensorimotor cortex. *J. Neurophysiol.* 79 1102–1107 9463466 10.1152/jn.1998.79.2.1102

[B53] Paus T. Sipila P. K. Strafella A. P. (2001). Synchronization of neuronal activity in the human primary motor cortex by transcranial magnetic stimulation: an EEG study. *J. Neurophysiol.* 86 1983–1990 11600655 10.1152/jn.2001.86.4.1983

[B54] Pfurtscheller G Lopes da Silva F. H. (1999). Event-related EEG/MEG synchronization and desynchronization: basic principles. *Clin. Neurophysiol.* 110 1842–1857 10.1016/S1388-2457(99)00141-8 10576479

[B55] Plewnia C. Rilk A. J. Soekadar S. R. Arfeller C. Huber H. S. Sauseng P. (2008). Enhancement of long-range EEG coherence by synchronous bifocal transcranial magnetic stimulation. *Eur. J. Neurosci.* 27 1577–1578 10.1111/j.1460-9568.2008.06124.x 18336566

[B56] Pratt K. L. Mattson R. H. Weikers N. J. Williams R. (1968). EEG activation of epileptics following sleep deprivation: a prospective study of 114 cases. *Electroencephalogr. Clin. Neurophysiol.* 24 11–15 10.1016/0013-4694(68)90061-8 4169743

[B57] Rosanova M. Casali A. Bellina V. Resta F. Mariotti M. Massimini M. (2009). Natural frequencies of human corticothalamic circuits. *J. Neurosci.* 29 7679–7685 10.1523/JNEUROSCI.0445-09.2009 19535579 PMC6665626

[B58] Rosenthal J. Waller H. J. Amassian V. E. (1967). An analysis of activation of motor cortical neurons by surface stimulation. *J. Neurophysiol.* 30 844–858 6035693 10.1152/jn.1967.30.4.844

[B59] Rossini P. M. Barker A. T. Berardelli A. Caramia M. D. Caruso G. Cracco R. Q. (1994). Non-invasive electrical and magnetic stimulation of the brain, spinal cord and roots: basic principles and procedures for routine clinical application. Report of an IFCN committee. *Electroencephalogr. Clin. Neurophysiol.* 91 79–92 10.1016/0013-4694(94)90029-9 7519144

[B60] Siegel M. Warden M. R. Miller E. K. (2009). Phase-dependent neuronal coding of objects in short-term memory. *Proc. Natl. Acad. Sci. U.S.A.* 106 21341–21346 10.1073/pnas.0908193106 19926847 PMC2779828

[B61] Silber M. H. Ancoli-Israel S. Bonnet M. H. Chokroverty S. Grigg-Damberger M. M. Hirshkowitz M. (2007). The visual scoring of sleep in adults. *J. Clin. Sleep Med.* 3 121–131 17557422

[B62] Smith C. MacNeill C. (1994). Impaired motor memory for a pursuit rotor task following stage 2 sleep loss in college students. *J. Sleep Res.* 3 206–213 10.1111/j.1365-2869.1994.tb00133.x 10607127

[B63] Stampi C. Stone P. Michimori A. (1995). A new quantitative method for assessing sleepiness: the Alpha Attenuation test. *Work Stress* 9 368–376 10.1080/02678379508256574

[B64] Steriade M Llinás R. R. (1988). The functional states of the thalamus and the associated neuronal interplay. *Physiol. Rev.* 68 649–742 2839857 10.1152/physrev.1988.68.3.649

[B65] Steriade M. (2003). The corticothalamic system in sleep. *Front. Biosci.* 8:878–899 10.2741/1043 12700074

[B66] Steriade M. (2005). Sleep, epilepsy and thalamic reticular inhibitory neurons. *Trends Neurosci.* 28 317–324 10.1016/j.tins.2005.03.007 15927688

[B67] Thut G. Miniussi C. (2009). New insights into rhythmic brain activity from TMS-EEG studies. *Trends Cogn. Sci.* 13 182–189 10.1016/j.tics.2009.01.004 19286414

[B68] Thut G. Northoff G. Ives J. R. Kamitani Y. Pfennig A. Kampmann F. (2003). Effects of single-pulse transcranial magnetic stimulation (TMS) on functional brain activity: a combined event-related TMS and evoked potential study. *Clin. Neurophysiol.* 114 2071–2080 10.1016/S1388-2457(03)00205-0 14580605

[B69] Thut G. Pascual-Leone A. (2010). A review of combined TMS-EEG studies to characterize lasting effects of repetitive TMS and assess their usefulness in cognitive and clinical neuroscience. *Brain. Topogr.* 22 219–232 10.1007/s10548-009-0115-4 19862614 PMC3260526

[B70] Tiitinen H. Virtanen J. Ilmoniemi R. J. Kamppuri J. Ollikainen M. Ruohonen J. (1999). Separation of contamination caused by coil clicks from responses elicited by transcranial magnetic stimulation. *Clin. Neurophysiol.* 110 982–985 10.1016/S1388-2457(99)00038-3 10400214

[B71] Van Der Werf Y. D. Sadikot A. F. Strafella A. P. Paus T. (2006). The neural response to transcranial magnetic stimulation of the human motor cortex. II Thalamocortical contributions. *Exp. Brain Res.* 175 246–255 10.1007/s00221-006-0548-x 16832683

[B72] Veniero D. Brignani D. Thut G. Miniussi C. (2011). Alpha-generation as basic response-signature to transcranial magnetic stimulation (TMS) targeting the human resting motor cortex: a TMS/EEG co-registration study. *Psychophysiology* 48 381–389 10.1111/j.1469-8986.2011.01218.x 21542853

[B73] Walker M. P. Brakefield T. Morgan A. Hobson J. A. Stickgold R. (2002a). Practice with sleep makes perfect: sleep-dependent motor skill learning. *Neuron* 35 205–211 10.1016/S0896-6273(02)00746-8 12123620

[B74] Walker M. P. Liston C. Hobson J. A. Stickgold R. (2002b). Cognitive flexibility across the sleep–wake cycle: REM-sleep enhancement of anagram problem solving. *Cogn. Brain Res*. 14 317–324 10.1016/S0926-6410(02)00134-9 12421655

